# Basolateral amygdala activation enhances object recognition memory by inhibiting anterior insular cortex activity

**DOI:** 10.1073/pnas.2203680119

**Published:** 2022-05-27

**Authors:** Yan-Fen Chen, Qi Song, Paola Colucci, Federica Maltese, Cristina Siller-Pérez, Karina Prins, James L. McGaugh, Erno J. Hermans, Patrizia Campolongo, Nael Nadif Kasri, Benno Roozendaal

**Affiliations:** ^a^Department of Cognitive Neuroscience, Radboud University Medical Center, 6500 HB Nijmegen, the Netherlands;; ^b^Donders Institute for Brain, Cognition and Behaviour, Radboud University, 6525 EN Nijmegen, the Netherlands;; ^c^Department of Physiology and Pharmacology, Sapienza University of Rome, 00185 Rome, Italy;; ^d^Istituto di Ricovero e Cura a Carattere Scientifico, Santa Lucia Foundation, 00179 Rome, Italy;; ^e^Emotional Brain Institute, The Nathan Kline Institute, Orangeburg, NY 10962;; ^f^Center for the Neurobiology of Learning and Memory, Department of Neurobiology and Behavior, University of California, Irvine, CA 92697-3800;; ^g^Department of Human Genetics, Radboud University Medical Center, 6500 HB Nijmegen, the Netherlands

**Keywords:** basolateral amygdala, norepinephrine, emotional arousal, insular cortex, salience network

## Abstract

Noradrenergic activation of the basolateral amygdala (BLA) by emotional arousal enhances different forms of recognition memory via functional interactions with the insular cortex (IC). Human neuroimaging studies have revealed that the anterior IC (aIC), as part of the salience network, is dynamically regulated during arousing situations. Emotional stimulation first rapidly increases aIC activity but suppresses it in a delayed fashion. Here, we investigated in male Sprague-Dawley rats whether the BLA influence on recognition memory is associated with an increase or suppression of aIC activity during the postlearning consolidation period. We first employed anterograde and retrograde viral tracing and found that the BLA sends dense monosynaptic projections to the aIC. Memory-enhancing norepinephrine administration into the BLA following an object training experience suppressed aIC activity 1 h later, as determined by a reduced expression of the phosphorylated form of the transcription factor cAMP response element-binding (pCREB) protein and neuronal activity marker c-Fos. In contrast, the number of perisomatic γ-aminobutyric acid (GABA)ergic inhibitory synapses per pCREB-positive neuron was significantly increased, suggesting a dynamic up-regulation of GABAergic tone. In support of this possibility, pharmacological inhibition of aIC activity with a GABAergic agonist during consolidation enhanced object recognition memory. Norepinephrine administration into the BLA did not affect neuronal activity within the posterior IC, which receives sparse innervation from the BLA. The evidence that noradrenergic activation of the BLA enhances the consolidation of object recognition memory via a mechanism involving a suppression of aIC activity provides insight into the broader brain network dynamics underlying emotional regulation of memory.

## Significance

The basolateral amygdala is known to mediate emotional arousal effects on different forms of recognition memory via functional interactions with the insular cortex. Human neuroimaging studies have revealed that emotional stimulation first rapidly increases activity of the anterior division of the insular cortex, as part of the large-scale salience network, but suppresses anterior insular cortex activity in a delayed fashion. Here, we show that the effect of noradrenergic activation of the basolateral amygdala on enhancing object recognition memory is associated with a suppression of anterior insular cortex activity during the postlearning consolidation period. Such delayed emotional arousal effects on brain network dynamics might disrupt attentional reorienting toward new external stimuli and thereby protect the ongoing consolidation process from interference.

Extensive evidence indicates that emotional arousal promotes the formation of long-term memory (1), which is vital for successful adaptation to both dangerous and favorable situations. Animal and human studies have shown that noradrenergic activation of the basolateral amygdala (BLA), induced by emotionally arousing stimulation (2), is crucially involved in enhancing long-term memory by influencing information storage processes involving other brain regions (3, 4). Noradrenergic activation of the BLA also enhances the consolidation of object recognition memory (5), which primarily relies on cortical structures (6, 7). There is extensive evidence that the insular cortex (IC) is involved in object recognition memory (6, 8, 9) as well as in other forms of recognition memory, such as conditioned taste aversion and facial and tactile recognition (10). Direct stimulation of transcriptional activity within the IC by either cAMP response element-binding (CREB) protein or histone acetylation enhances consolidation processes of object recognition memory and conditioned taste aversion (9, 11, 12), whereas a local suppression of protein synthesis impairs recognition memory (6). The BLA is also densely interconnected with the IC (13, 14), and several studies have indicated a functional crosstalk between these two brain regions in mediating emotional arousal effects on recognition memory (12, 15).

Findings of human studies using functional MRI have indicated that the BLA and anterior division of the IC (aIC) are key nodes of a large-scale “salience” network (16) and that activity of this brain network is dynamically regulated during and after processing of emotionally arousing information (17). Emotionally arousing events, via noradrenergic activation, first rapidly increase aIC activity and its functional connectivity with other regions of the salience network (18–20), inducing a hypervigilant state and prioritized encoding of emotionally arousing information (21). However, in a delayed period after the initial arousal exposure, the aIC and salience network activity shuts off (17, 22), which may enable the activation of other brain networks and enhancement of higher-order cognitive processes, including long-term memory storage (17). In support of these findings, animal studies have shown that memory-enhancing adrenergic or glucocorticoid stress hormone administration after training on object recognition or inhibitory avoidance tasks induces a delayed inhibition of neuronal activity within the aIC (23, 24). However, prior studies have not investigated whether a suppression of aIC activity during the consolidation phase contributes to emotionally influenced memory enhancement and whether noradrenergic activation of the BLA is able to induce such delayed suppression of aIC neural activity. Moreover, although some findings suggest that the aIC and posterior IC (pIC) are implicated in different neural functions (25, 26), it is not known whether the BLA differentially interacts with these two subareas in regulating emotional arousal effects on recognition memory.

In the current study, we first employed anterograde and retrograde viral tracing to examine the pattern of BLA projections to the aIC and pIC. We found that the BLA sends dense monosynaptic projections to the aIC and only sparse projections to the pIC. Then, we administered norepinephrine (NE) into the BLA immediately following an object training experience to identify resulting changes in neuronal activity within the aIC and pIC. The memory-enhancing NE treatment selectively reduced neuronal activity within the aIC during the postlearning consolidation period, whereas neuronal activity within the pIC was not affected. Most importantly, we found that such inhibition of the aIC was sufficient to enhance the consolidation of object recognition memory.

## Results

### Noradrenergic Activation of the BLA Enhances Object Recognition Memory.

**Fig. 1.**
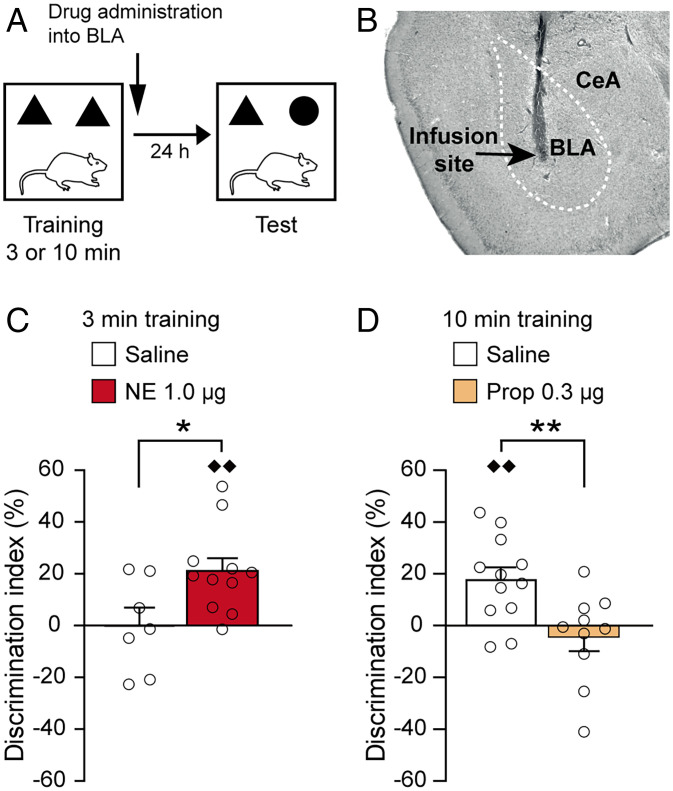
Noradrenergic activation of the BLA enhances object recognition memory. (*A*) Experimental design of the object recognition task. (*B*) Representative photomicrograph illustrating an infusion needle tip within the BLA. Arrow points to the needle tip. CeA, central amygdala. (*C*) NE (1.0 μg in 0.2 μL) administered bilaterally into the BLA immediately after a 3-min training trial enhanced 24-h object recognition memory. Data are presented as discrimination index (mean ± SEM; see *Materials and Methods*). Dots represent individual data points (saline: *n* = 7 rats; NE: *n* = 11 rats). *P < 0.05 versus saline; ♦♦*P* < 0.01 versus chance. (*D*) The β-adrenoceptor antagonist propranolol (Prop; 0.3 μg in 0.2 μL) administered into the BLA immediately after a 10-min training trial impaired 24-h object recognition memory. Data are presented as discrimination index (mean ± SEM) (saline: *n* = 12 rats; propranolol: *n* = 10 rats). **P < 0.01 versus saline; ♦♦*P* < 0.01 versus chance.

To examine the effect of noradrenergic activation of the BLA on object recognition memory, male Sprague-Dawley rats were trained on an object recognition task for 3 min and immediately after the training trial given bilateral microinfusions of NE (1.0 μg in 0.2 μL) or saline into the BLA. This short training session is not sufficient to induce long-term memory for the object (5). Other rats were trained for 10 min, which induces robust long-term memory (5), followed by bilateral administration of the β-adrenoceptor antagonist propranolol (0.3 μg in 0.2 μL) or saline into the BLA. The drug treatment groups did not differ in total exploration time of the two objects during the training trial (*P* values > 0.08; *SI Appendix*, Fig. S1). To determine whether animals exhibit a long-term memory for the object seen during the training, they were given a 24-h retention test in which one object was familiar and the other object was novel. Fig. 1*A* depicts the experimental protocol, and Fig. 1*B* shows a representative photomicrograph of an infusion needle tip terminating within the BLA.

As shown in Fig. 1*C*, NE administration into the BLA immediately after a 3-min training trial enhanced 24-h object recognition memory (*SI Appendix*, Fig. S2*A* for infusion sites). As expected, the discrimination index of saline-treated control rats did not differ from zero (i.e., chance level) (one-sample *t* test: *t*_6_ = −0.001, *P* = 1.00), indicating that they did not show any evidence of retention. Posttraining NE administration into the BLA significantly enhanced the discrimination index (unpaired *t* test: *t*_16_ = −2.55, *P* = 0.02, compared to saline). Further, the discrimination index of NE-treated rats was significantly greater than zero (*t*_10_ = 4.21, *P* = 0.002), indicating that they exhibited a significant exploration preference for the novel object. The NE and saline groups did not differ in total exploration time of the two objects during the retention test (*t*_16_ = −0.83, *P* = 0.42; *SI Appendix*, Fig. S1), indicating that the drug treatment did not induce a general change in the rats’ incentive to explore the objects.

Conversely, propranolol administration into the BLA to suppress endogenous noradrenergic activity after a 10-min training trial impaired 24-h object recognition memory (Fig. 1*D* and *SI Appendix*, Fig. S2*B* for infusion sites). After this more extensive training, the discrimination index of saline-treated rats was significantly greater than zero (one-sample *t* test: *t*_11_ = 3.72, *P* = 0.003). Posttraining propranolol significantly impaired the discrimination index on the 24-h retention test (*t*_19_ = 2.80, *P* = 0.01), which did not differ from zero (one-sample *t* test: *t*_9_ = −0.78, *P* = 0.46). The propranolol and saline groups did not differ in total exploration time of the two objects during the retention test (*t*_20_ = 1.80, *P* = 0.09; *SI Appendix*, Fig. S1).

### Noradrenergic Activation of the aIC, but Not pIC, Enhances Object Recognition Memory.

**Fig. 2.**
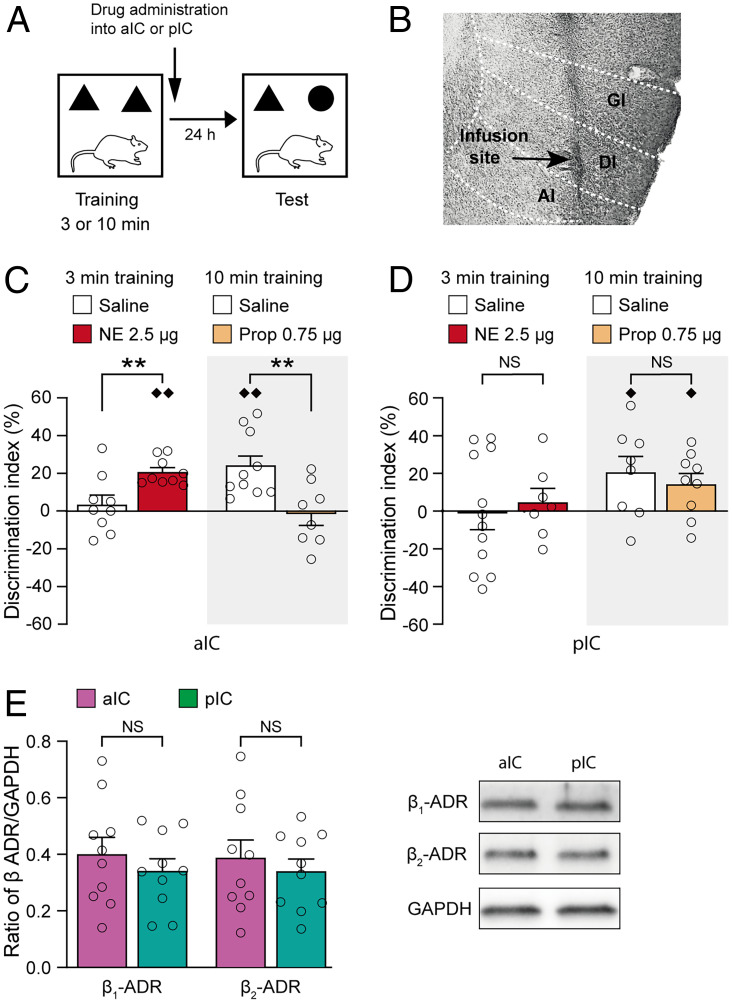
Noradrenergic activation of the aIC, but not the pIC, enhances object recognition memory. (*A*) Experimental design of the object recognition task. (*B*) Representative photomicrograph illustrating an infusion needle tip within the aIC. Arrow points to the needle tip. Dashed lines indicate the different subdivisions of the aIC. GI, granular IC; DI, dysgranular IC; AI, agranular IC. (*C*) NE (2.5 μg in 0.5 μL) administered bilaterally into the aIC immediately after a 3-min training trial enhanced 24-h object recognition memory, whereas the β-adrenoceptor antagonist propranolol (Prop; 0.75 μg in 0.5 μL) administered after a 10-min training trial impaired 24-h object recognition memory. Data are presented as discrimination index (mean ± SEM; see *Materials and Methods*). Dots represent individual data points (saline–3 min: *n* = 9 rats; NE–3 min: *n* = 9 rats; saline–10 min: *n* = 10 rats; propranolol–10 min: *n* = 8 rats). **P < 0.01 versus saline; ♦♦*P* < 0.01 versus chance. (*D*) NE (2.5 g in 0.5 μL) or propranolol (0.75 μg in 0.5 μL) administration into the pIC did not affect 24-h object recognition memory. Data are presented as discrimination index (mean ± SEM) (saline–3 min: *n* = 12 rats; NE–3 min: *n* = 7 rats; saline–10 min: *n* = 8 rats; propranolol–10 min: *n* = 9 rats). ♦*P* < 0.05 versus chance. NS, not significant. (*E*) β_1_-adrenoceptor (ADR) and β_2_-adrenoceptor protein levels did not differ between the aIC and pIC. *Left*: β-adrenoceptor/GAPDH ratio (mean ± SEM) within the aIC and pIC (*n* = 10 rats). NS, not significant. *Right*: representative Western blot images.

Next, we investigated the effects of NE and propranolol administration into either the aIC or pIC on object recognition memory (*SI Appendix*, Fig. S3 for infusion sites). Fig. 2*A* depicts the experimental protocol, and Fig. 2*B* shows a representative photomicrograph of an infusion needle tip terminating within the aIC.

As shown in Fig. 2*C*, the discrimination index of rats administered saline into the aIC after a 3-min training trial did not differ from zero on a 24-h retention test (one-sample *t* test: *t*_8_ = 0.65, *P* = 0.53). NE (2.5 μg in 0.5 μL) administration into the aIC significantly enhanced the discrimination index (unpaired *t* test: *t*_16_ = −3.10, *P* = 0.007). Further, the discrimination index of NE-treated rats was significantly greater than zero (*t*_8_ = 8.79, *P* < 0.0001). Conversely, propranolol (0.75 μg in 0.5 μL) administration into the aIC after a 10-min training trial significantly impaired the discrimination index on a 24-h retention test (*t*_16_ = 3.23; *P* = 0.005). After this more extensive training, the discrimination index of rats administered saline into the aIC was significantly greater than zero (*t*_9_ = 4.55, *P* = 0.001), whereas that of rats administered propranolol into the aIC did not differ from zero (*t*_7_ = 0.25, *P* = 0.81). The drug treatment groups did not differ in total exploration time of the two objects during the training or retention test (*P* values > 0.14; *SI Appendix*, Fig. S4).

Unlike drug infusions into the aIC, NE or propranolol administration into the pIC did not affect the discrimination index on a 24-h retention test (unpaired *t* tests, NE: *t*_17_ = 0.45, *P* = 0.66; propranolol: *t*_15_ = 0.64, *P* = 0.53; Fig. 2*D*). The drug treatment groups also did not differ in total exploration time of the two objects during the training or retention test (*P* values > 0.09, *SI Appendix*, Fig. S4).

Western blot experiments indicated that protein levels of both the β_1_-adrenoceptor (paired *t* test: *t*_9_ = 1.00, *P* = 0.34) and β_2_-adrenoceptor (*t*_9_ = 0.86, *P* = 0.41) did not differ between the aIC and the pIC (Fig. 2*E*). Thus, this selective involvement of the aIC, and not the pIC, in mediating noradrenergic effects on object recognition memory cannot be explained by a difference in receptor expression.

### Dense Structural Connections from the BLA to the aIC, but Not the pIC.

**Fig. 3.**
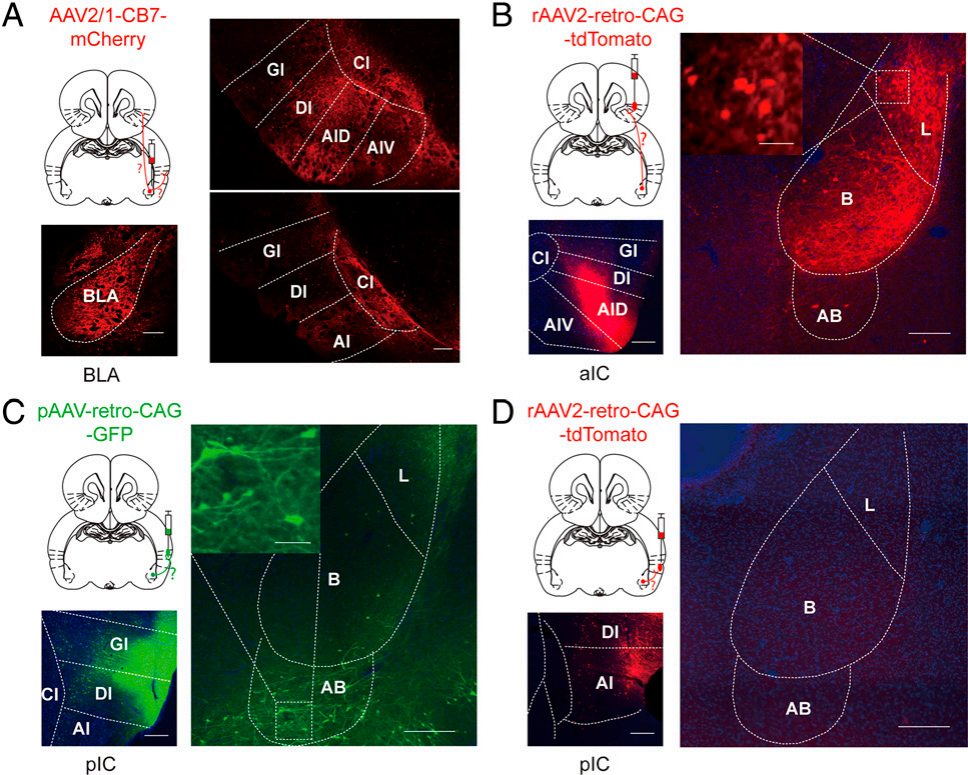
Dense structural connections from the BLA to the aIC, but not the pIC. (*A*) AAV2/1-CB7-mCherry anterograde virus injected into the BLA induced dense labeling of axon terminals within the aIC. The diagram depicts the injection site and the anatomical projection (red line) being investigated. *Left*: example image of injection site in the BLA. *Top right*: example image demonstrating mCherry-positive axon terminals (red) in the agranular and dysgranular aIC. *Bottom right*: example image demonstrating only a few mCherry-positive axon terminals in the pIC. AI, agranular IC; AID, agranular IC, dorsal part; AIV, agranular IC, ventral part Cl, claustrum; DI, dysgranular IC; GI, granular IC. (*B*) AAV2-retro-CAG-tdTomato retrograde virus injected into the aIC induced dense labeling of cell bodies within the lateral and basal nuclei of the BLA and sparse labeling within the accessory basal nucleus. The diagram depicts the injection site and the anatomical projection (red line) being investigated. *Left*: example image of injection site in the agranular and dysgranular aIC. *Right*: example image demonstrating tdTomato-positive cell bodies (red) in the BLA. *Inset*: tdTomato-labeled neurons. AB, accessory basal nucleus; B, basal nucleus; L, lateral nucleus. (*C*) pAAV-retro-CAG-GFP retrograde virus injected into the granular and dysgranular pIC induced only sparse labeling of cell bodies within the BLA. The diagram depicts the injection site and the anatomical projection (green line) being investigated. *Left*: example image of injection site in the granular and dysgranular pIC. *Right*: example image demonstrating sparse GFP-positive cell bodies (green) in the accessory basal nucleus. *Inset*: GFP-labeled neurons. (*D*) AAV2-retro-CAG-tdTomato retrograde virus injected into the agranular pIC induced no visible labeling of neurons in the BLA. The diagram depicts the injection site and the anatomical projection (red line) being investigated. *Left*: example image of injection site in the agranular pIC. *Right*: example image showing no tdTomato-positive neurons (red) in the BLA (Scale bars, 250 μm; *Inset*, 50 μm).

Then, we investigated whether the BLA might preferentially project to the aIC. We injected the anterograde virus AAV2/1-CB7-mCherry (27) into the BLA (Fig. 3*A*) and examined the distribution of labeled axon terminals within the aIC and pIC. We found dense labeling of axon terminals within the agranular and dysgranular subdivisions of the aIC and only sparse labeling of axon terminals within the pIC (Fig. 3*A*). To confirm the preferential projections from the BLA to the aIC, we injected a retrograde virus into either the aIC or the pIC. Injection of AAV2-retro-CAG-tdTomato (28) into the agranular and dysgranular subdivisions of the aIC resulted in dense labeling of cell bodies within both the lateral and basal nuclei of the BLA and sparse labeling of cell bodies within the accessory basal nucleus (Fig. 3*B*). In contrast, injection of pAAV-retro-CAG-GFP into the granular and dysgranular pIC resulted in very few labeled cell bodies within the lateral and basal nuclei of the BLA and moderate labeling within the accessory basal nucleus (Fig. 3*C*). AAV2-retro-CAG-tdTomato injected into the agranular pIC did not result in any visible labeling of cell bodies within the BLA (Fig. 3*D*). These findings thus indicate that the BLA sends dense monosynaptic projections to the aIC and only sparse projections to the pIC.

### Noradrenergic Activation of the BLA Reduces Expression of the Phosphorylated (i.e., Activated) Form of the Transcription Factor CREB (pCREB) within the aIC.

**Fig. 4.**
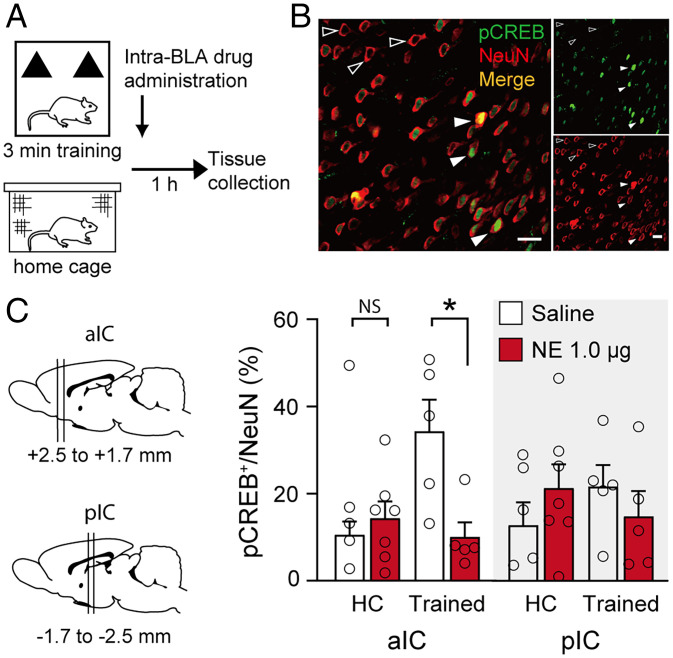
Noradrenergic activation of the BLA reduces pCREB expression within the aIC during the postlearning consolidation period. (*A*) Experimental protocol. Rats were trained for 3 min on an object recognition task followed immediately by NE (1.0 μg in 0.2 μL) or saline administration into the BLA. Other rats received NE or saline infusions without training (HC). One hour later, rats were perfused for immunofluorescence of the transcription factor pCREB and neuronal marker NeuN. (*B*) Representative images of double staining of pCREB (green) and NeuN (red). Filled arrowheads point to pCREB-positive neurons, unfilled arrowheads point to pCREB-negative neurons (Scale bar, 20 μm). (*C*) Posttraining NE administration into the BLA selectively reduced the pCREB/NeuN ratio (mean ± SEM) within the aIC (AP, +2.5 to +1.7 mm) and not the pIC (AP, −1.7 to −2.5 mm). Dots represent individual data points (saline–trained: *n* = 5 rats; NE–trained: *n* = 5 rats; saline–HC: *n* = 4 rats; NE–HC: *n* = 7 rats). *P < 0.05. NS, not significant.

Given this selective pattern of BLA projections to the aIC, we examined whether NE (1.0 μg in 0.2 μL) administration into the BLA after a 3-min object training experience also selectively triggers neuronal activity changes within the aIC. For this, we determined the number of neurons that showed immunofluorescence for pCREB (29), normalized for the total number of neurons (i.e., pCREB/NeuN (Neuronal Nuclei) ratio), within cortical layers II/III of the aIC [anteroposterior (AP), +2.5 to +1.7 mm] and pIC (AP, −1.7 to −2.5 mm) 1 h after the training and posttraining drug treatment (Fig. 4 *A–C*) (*SI Appendix*, Table S1 for training data). Additional home-cage (HC) control rats received NE or saline infusions into the BLA without training.

The effect of NE administration into the BLA on the number of pCREB-positive neurons within the aIC and pIC was analyzed by using a mixed-model ANOVA with the between-subject factors NE treatment (NE versus saline) and training (trained versus HC) and the within-subject factor IC subarea (aIC versus pIC) (Fig. 4*C*). We found no main effects of NE treatment (*F*_1,17_ = 1.03, *P* = 0.32), training (*F*_1,17_ = 1.39, *P* = 0.26), or subarea (*F*_1,17_ = 0.02, *P* = 0.90), but did find a significant NE treatment × subarea interaction effect (*F*_1,17_ = 4.76, *P* = 0.04). In a next step, we analyzed the effects of NE treatment and training on the number of pCREB-positive neurons within the aIC and pIC separately. Within the aIC, we found a significant NE treatment effect (*F*_1,17_ = 4.47, *P* = 0.049) as well as a significant NE treatment × training interaction effect (*F*_1,17_ = 8.41, *P* = 0.01) and a near significant effect of training (*F*_1,17_ = 4.05, *P* = 0.06; Fig. 4*C*). Post hoc analyses indicated that NE administration into the BLA of trained rats significantly reduced the number of pCREB-positive neurons within the aIC (*t*_8_ = 3.04, *P* = 0.02), whereas NE administration into the BLA of HC rats did not reduce the number of pCREB-positive neurons within the aIC (*t*_9_ = −0.67, *P* = 0.52). In contrast, NE treatment or training did not significantly affect the number of pCREB-positive neurons within the pIC (NE treatment: *F*_1,17_ = 0.02, *P* = 0.89; training: *F*_1,17_ = 0.04, *P* = 0.84; NE treatment × training: *F*_1,17_ = 1.82, *P* = 0.20; Fig.4*C*).

In saline-treated rats, the training experience itself increased the number of pCREB-positive neurons within the aIC (*t*_7_ = 2.76, *P* = 0.03, compared to saline-treated HC rats). However, as this 3-min training session is too brief to induce any 24-h memory, we also examined the impact of a more extensive 10-min training session, which, like NE treatment, induces robust 24-h memory (*SI Appendix*, Table S2 for training data). HC rats received no training. As shown in *SI Appendix*, Fig. S5, neither the 3-min nor the 10-min training session reduced the number of pCREB-positive neurons within the aIC 1 h later (one-way ANOVA: *F*_2,21_ = 0.10, *P* = 0.90). This finding thus indicates that posttraining noradrenergic activation, and not memory induction per se, is associated with a reduction in pCREB activity within the aIC.

### Noradrenergic Activation of the BLA Reduces Coexpression of c-Fos and pCREB within the aIC.

**Fig. 5.**
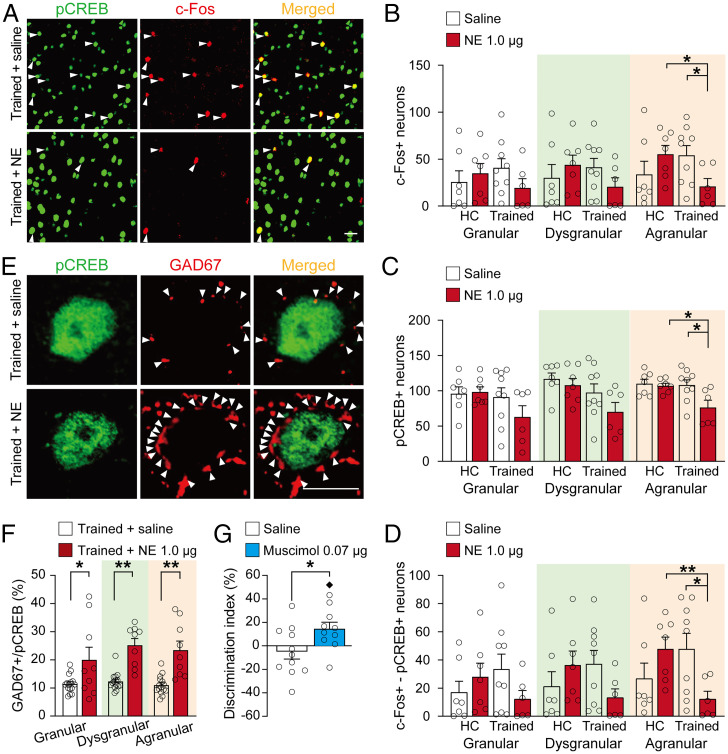
Noradrenergic activation of the BLA increases GABAergic inhibition within the aIC during the postlearning consolidation period. (*A*) Representative images showing immunofluorescence of c-Fos (red) and pCREB (green). Arrowheads point to neurons showing coexpression of c-Fos and pCREB (Scale bar, 20 μm). (*B–D*) The number of nuclei showing expression of c-Fos (*B*) and pCREB (*C*) and colocalization of c-Fos and pCREB (*D*) (mean ± SEM) within the different aIC subdivisions. Dots represent individual data points (saline–trained: *n* = 9 rats; NE–trained: *n* = 6 rats; saline–HC: *n* = 9 rats; NE–HC: *n* = 6 rats). *P < 0.05, **P < 0.01. (*E*) Representative images showing immunofluorescence of GAD67-positive puncta (red) and pCREB (green) within the aIC. Arrowheads point to GAD67-positive puncta (Scale bar, 10 μm). (*F*) The number of GAD67-positive puncta/pCREB-positive nucleus (mean ± SEM) within the different aIC subdivisions. Dots represent individual data points (saline: *n* = 15 images, from five rats; NE: *n* = 9 images, from three rats). *P < 0.05, **P < 0.01 versus saline. (*G*) The GABAergic agonist muscimol (0.07 μg in 0.5 μL) administered bilaterally into the aIC 1 h after a 3-min training trial enhanced 24-h object recognition memory. Data are presented as discrimination index (mean ± SEM). Dots represent individual data points (saline: *n* = 11 rats; muscimol: *n* = 10 rats). *P < 0.05 versus saline; ♦*P* < 0.05 versus chance.

Our finding that posttraining NE administration into the BLA reduced the number of pCREB-positive neurons within the aIC could reflect either an overall inhibition of transcriptional activity or, alternatively, an increased signal-to-noise ratio such that the remaining pCREB-positive neurons are more actively engaged in local network activity (11). To address this question, we examined, in different groups of animals, whether NE administration into the BLA of trained rats or HC control rats altered the number of cells expressing the neuronal activity marker c-Fos (30) as well as those showing coexpression of c-Fos with pCREB (Fig. 5*A*). c-Fos is an immediate early gene product that is a well-established molecular marker for identifying recently activated cells (31). For this experiment, we examined immunofluorescence within cortical layers II/III of each of the three main subdivisions of the aIC 1 h after the training and posttraining drug treatment (*SI Appendix*, Table S3 for training data).

The effect of NE administration into the BLA on the number of c-Fos–positive neurons within the aIC was analyzed by using a mixed-model ANOVA with the between-subject factors NE treatment (NE versus saline) and training (trained versus HC) and the within-subject factor aIC subdivision (agranular, dysgranular, versus granular) (Fig. 5*B*). We found no main effects of NE treatment (*F*_1,25_ = 0.24, *P* = 0.63) or training (*F*_1,25_ = 0.18, *P* = 0.68) but did reveal a significant aIC subdivision effect (*F*_2,50_ = 8.62, *P* = 0.01) and an almost significant NE treatment × training × subdivision interaction effect (*F*_2,50_ = 2.95, *P* = 0.06). In a next step, we analyzed the effects of NE treatment and training on the number of c-Fos–positive neurons within the three aIC subdivisions separately. Within the agranular aIC, two-way ANOVA indicated no main effects of NE treatment (*F*_1,25_ = 0.05, *P* = 0.87) or training (*F*_1,25_ = 0.07, *P* = 0.84) but did reveal a significant NE treatment × training interaction effect (*F*_1,25_ = 6.16, *P* = 0.02). Post hoc analyses indicated that NE administration into the BLA of trained rats significantly reduced the number of c-Fos–positive neurons within the agranular aIC compared to both saline-treated trained rats (*t*_13_ = 2.32, *P* = 0.04) and NE-treated HC rats (*t*_11_ = 2.69, *P* = 0.02; Fig. 5*B*), whereas NE administration into the BLA of HC rats did not reduce the number of c-Fos–positive neurons (*t*_12_ = 1.27, *P* = 0.23, compared to saline-treated HC rats). The training experience of saline-treated rats did not significantly alter the number of c-Fos–positive neurons within the agranular aIC (*t*_14_ = 1.21, *P* = 0.24). NE treatment or training did not significantly affect the number of c-Fos–positive neurons within the granular aIC (NE treatment: *F*_1,25_ = 0.16, *P* = 0.76; training: *F*_1,25_ = 0.0002, *P* = 0.99; NE treatment × training: *F*_1,25_ = 2.04, *P* = 0.17) or dysgranular aIC (NE treatment: *F*_1,25_ = 0.04, *P* = 0.88; training: *F*_1,25_ = 0.12, *P* = 0.79; NE treatment × training: *F*_1,25_ = 2.40, *P* = 0.13).

Analysis of the effect of NE administration into the BLA on the number of pCREB-positive neurons within the three aIC subdivisions indicated no main effect of NE treatment (*F*_1,25_ = 2.88, *P* = 0.10) but did reveal significant effects of training (*F*_1,25_ = 5.17, *P* = 0.03) and aIC subdivision (*F*_2,50_ = 6.64, *P* = 0.003; Fig. 5*C*). No significant interaction effects were found for NE treatment × training (*F*_1,25_ = 1.85, *P* = 0.18), NE treatment × subdivision (*F*_2,50_ = 0.26, *P* = 0.77), or NE treatment × training × subdivision (*F*_2,50_ = 0.35, *P* = 0.71). Follow-up analyses examined NE treatment and training effects on the number of pCREB-positive neurons within the three aIC subdivisions separately. Within the agranular aIC, two-way ANOVA indicated main effects of NE treatment (*F*_1,25_ = 5.00, *P* = 0.03) and training (*F*_1,25_ = 5.68, *P* = 0.02) and an almost significant NE treatment × training interaction effect (*F*_1,25_ = 3.84, *P* = 0.06). Post hoc analyses indicated that NE administration into the BLA of trained rats significantly reduced the number of pCREB-positive neurons within the agranular aIC compared to both saline-treated trained rats (*t*_13_ = 2.54, *P* = 0.02) and NE-treated HC rats (*t*_11_ = 3.00, *P* = 0.01; Fig. 5*C*), whereas NE administration into the BLA of HC rats did not reduce the number of pCREB-positive neurons (*t*_12_ = 0.43, *P* = 0.68, compared to saline-treated HC rats). The training experience of saline-treated rats did not significantly alter the number of pCREB-positive neurons within the agranular aIC (*t*_14_ = 0.60, *P* = 0.56). NE treatment or training did not significantly affect the number of pCREB-positive neurons within the granular aIC (NE treatment: *F*_1,25_ = 1.13, *P* = 0.30; training: *F*_1,25_ = 0.12, *P* = 0.79; NE treatment × training: *F*_1,25_ = 2.40, *P* = 0.13). Within the dysgranular aIC, we found no NE treatment (*F*_1,25_ = 2.49, *P* = 0.13) or NE treatment × training interaction effect (*F*_1,25_ = 0.65, *P* = 0.43), but we did find a significant effect of training (*F*_1,25_ = 6.23, *P* = 0.02).

Then, we determined the number of neurons that showed colocalized activity of c-Fos and pCREB to examine whether NE administration into the BLA changed neuronal activity in this subset of pCREB-positive neurons (Fig. 5*D*). We found no main effects of NE treatment (*F*_1,25_ = 0.36, *P* = 0.55) or training (*F*_1,25_ = 0.14, *P* = 0.71) but did reveal a significant aIC subdivision effect (*F*_2,50_ = 10.39, *P* < 0.0001) as well as significant NE treatment × training (*F*_1,25_ = 5.37, *P* = 0.03) and NE treatment × training × aIC subdivision interaction effects (*F*_2,50_ = 3.34, *P* = 0.04). Follow-up analyses examined NE treatment and training effects on the number of neurons coexpressing c-Fos and pCREB within the three aIC subdivisions separately. Within the agranular aIC, two-way ANOVA indicated no significant NE treatment (*F*_1,25_ = 0.53, *P* = 0.47) or training effect (*F*_1,25_ = 0.52, *P* = 0.48) but did reveal a significant NE treatment × training interaction effect (*F*_1,25_ = 8.12, *P* = 0.009). As shown in Fig. 5*D,* NE administration into the BLA of trained rats significantly reduced the number of neurons coexpressing c-Fos and pCREB within the agranular aIC compared to both saline-treated trained rats (*t*_13_ = 2.50, *P* = 0.03) and NE-treated HC rats (*t*_11_ = 3.41, *P* = 0.006), whereas NE administration into the BLA of HC rats did not reduce the number of neurons coexpressing c-Fos and pCREB (*t*_12_ = 1.52, *P* = 0.15, compared to saline-treated HC rats). The training experience of saline-treated rats did not significantly alter the number of neurons coexpressing c-Fos and pCREB within the agranular aIC (*t*_14_ = 1.34, *P* = 0.20). NE treatment or training did not significantly affect the number of neurons coexpressing c-Fos and pCREB within the granular aIC (NE treatment: *F*_1,25_ = 0.30, *P* = 0.59; training: *F*_1,25_ = 0.002, *P* = 0.97; NE treatment × training: *F*_1,25_ = 3.01, *P* = 0.10) or dysgranular aIC (NE treatment: *F*_1,25_ = 0.20, *P* = 0.66; training: *F*_1,25_ = 0.14, *P* = 0.72; NE treatment × training: *F*_1,25_ = 4.15, *P* = 0.052).

Thus, these findings show that NE administration into the BLA induces a training-specific reduction in the number of transcriptionally active cells within the aIC during the postlearning consolidation phase. This effect was only significant within the agranular subdivision of the aIC.

### Noradrenergic Activation of the BLA Increases GABAergic Inhibition within the aIC.

Next, we investigated whether this reduced number of pCREB-positive cells within the aIC reflects a change in excitatory or inhibitory activity. Therefore, we performed double staining for pCREB with glutamic acid decarboxylase 67 (GAD67), a marker for γ-aminobutyric acid (GABA)ergic inhibitory neurons (32). We found that pCREB was predominantly (>95%) expressed in excitatory (i.e., GAD67-negative) neurons and that NE administration into the BLA did not significantly change the ratio of pCREB-positive excitatory versus inhibitory cells within the different subdivisions of the aIC (NE treatment: *F*_1,12_ = 2.25, *P* = 0.16; subdivision: *F*_2,24_ = 2.67, *P* = 0.09; NE treatment × subdivision: *F*_2,24_ = 2.00, *P* = 0.16; *SI Appendix*, Fig. S6).

Then, we quantified the number of GAD67-positive puncta, as a measure of perisomatic inhibitory synapse contacts, that were localized at the somatic circumference of pCREB-positive neurons (33, 34) (Fig. 5*E* and *SI Appendix*, Table S4 for training data). Mixed-model ANOVA indicated a significant NE treatment effect (*F*_1,22_ = 32.74, *P* < 0.0001) but no aIC subdivision effect (*F*_2,44_ = 1.42, *P* = 0.25) or NE treatment × aIC subdivision effect (*F*_2,44_ = 0.86, *P* = 0.43; Fig. 5*F*). Post hoc analysis indicated that posttraining NE administration into the BLA significantly increased the number of GAD67-positive puncta per pCREB-positive neuron within the granular (*t*_22_ = −2.37, *P* = 0.03), dysgranular (*t*_22_ = −6.13, *P* < 0.0001), and agranular aIC (*t*_22_ = −4.59, *P* < 0.0001). These findings strongly suggest that posttraining NE administration into the BLA reduces neuronal activity of the aIC by increasing the level of GABAergic inhibitory tone.

### GABAergic Inhibition of the aIC Enhances Object Recognition Memory.

Lastly, we determined whether this increased GABAergic inhibition within the aIC during the postlearning consolidation period contributes to enhancement of object recognition memory. We trained rats on the object recognition task for 3 min and 1 h later administered the GABAergic agonist muscimol (0.07 μg in 0.5 μL) or saline bilaterally into the aIC (*SI Appendix*, Fig. S7 for infusion sites). As shown in Fig. 5*G*, muscimol administration into the aIC significantly enhanced the discrimination index at a 24-h retention test (unpaired *t* test: *t*_19_ = −2.27, *P* = 0.03). Further, the discrimination index of muscimol-treated rats was significantly greater than zero (one-sample *t* test: *t*_9_ = 2.60, *P* = 0.03), whereas that of saline-treated rats did not differ from zero (*t*_10_ = 0.77, *P* = 0.46). The muscimol and saline groups did not differ in total exploration time of the two objects during the training or retention test (*P* values > 0.19, *SI Appendix*, Table S5). Thus, these findings indicate that an inhibition of aIC activity during the consolidation period is sufficient to enhance object recognition memory.

## Discussion

These experiments investigated how noradrenergic activation of the BLA interacts with the aIC and pIC in mediating emotional arousal effects on enhancement of object recognition memory. Extensive prior findings indicate that noradrenergic activation of the BLA enhances memory consolidation by facilitating neural and synaptic plasticity mechanisms within its target regions (3, 4). Human neuroimaging studies have shown that arousing stimulation inhibits aIC activity during the postlearning consolidation period (17, 22), but previous studies have not investigated whether such suppression of aIC activity actively contributes to emotional memory enhancement. We found that the BLA sends dense monosynaptic projections to the aIC and that posttraining NE administration into the BLA induced a reduction in aIC activity 1 h later, an effect likely associated with an increased GABAergic inhibitory tone. Importantly, direct pharmacological inhibition of the aIC with the GABAergic agonist muscimol during the consolidation phase was sufficient to enhance object recognition memory. These findings thus provide evidence that noradrenergic activity within the BLA–aIC circuit enhances object recognition memory via a mechanism involving a reduction of aIC activity during the consolidation phase and thus yield insight into the broader regulation of emotional arousal effects on brain network dynamics.

The BLA is known to regulate different forms of recognition memory via functional interactions with the IC (12, 15, 24, 35). The IC, however, is a large and heterogeneous brain region (26, 36–38), and findings of both animal and human studies suggest that the aIC and pIC might be involved in regulating different functions (25, 37–39). Findings of lesion studies in rodents have indicated that the aIC is necessary for the acquisition of both conditioned taste aversion and water-maze learning (25) and that the pIC has a critical role in the consolidation and extinction of fear-based learning (40). A possible differential involvement of these two subareas in object recognition memory remained elusive. We found that NE or propranolol infusions into the aIC enhanced or impaired the consolidation of object recognition memory, respectively, whereas similar infusions into the pIC were ineffective. To investigate the anatomical projections from the BLA to these two IC subareas, we employed anterograde and retrograde viral tracing, which permits efficient and reliable access to projection neurons (27, 28). We found that both the basal and lateral nuclei of the BLA, which are the main input sites for sensory information (41), send dense monosynaptic projections to the agranular and dysgranular aIC, but only sparse projections to the pIC. The accessory basal nucleus was found to be predominantly connected to the pIC. Previous findings have shown that the accessory basal nucleus, like the pIC, is involved in regulating anxiety and fear-related behaviors (42). Our findings are comparable to those of a recent study that mapped the whole-brain connectivity of the mouse aIC and pIC (14).

Consistent with the evidence of this structural connectivity, we found that NE administration into the BLA after object training selectively altered neuronal activity within the aIC and not the pIC. The effect appears to be the greatest within the agranular subdivision of the aIC, which is considered a higher-order multimodal cortical region (43), and is thought to maintain cognitive representations of interoceptive states associated with previous experiences (44). In contrast to the predominantly excitatory effect of BLA activation on hippocampal activity (45, 46), we found that memory-enhancing NE administration into the BLA induced a reduction in the number of pCREB-positive cells within the aIC 1 h later. As the large majority (>95%) of these pCREB-expressing neurons were excitatory neurons, our findings suggest that NE administration inhibited neuronal excitability of the aIC. Consistent with these findings, we previously reported that posttraining NE administration into the BLA also induced a training-specific reduction in the acetylation levels of histone molecules within the aIC (24), which critically interact with pCREB in influencing transcriptional activity and memory (47). Future studies could use chemogenetic approaches to determine whether the monosynaptic projection from the BLA to the aIC mediates this NE effect on enhancing object recognition memory and reducing aIC activity during the postlearning consolidation period.

Previous findings have indicated that CREB overexpression within the aIC regulates memory allocation and that IC neurons with higher CREB levels are more likely to be recruited into encoding and storing recognition memory (11). Moreover, posttraining administration of a histone deacetylase inhibitor into the aIC was found to enhance object recognition memory (9, 12), whereas suppression of protein synthesis within the aIC impaired object recognition memory (6). Our finding of a reduction in pCREB expression might reflect an altered signal-to-noise ratio, such that the remaining pCREB-expressing neurons are more actively involved in local network activity. Thus, we also examined whether these remaining pCREB-expressing neurons showed higher expression levels of the neuronal activity marker c-Fos. NE administration, however, also induced a reduction in the number of c-Fos–expressing cells as well as the number of cells that showed coexpression of pCREB and c-Fos. These findings thus indicate that noradrenergic activation of the BLA reduces aIC activity during the postlearning consolidation period.

We recently reported that the effect of glucocorticoid administration into the prefrontal cortex on enhancing object recognition memory was also associated with a reduction in c-Fos expression within the aIC (48). Most importantly, suppression of noradrenergic activity of the BLA by propranolol blocked the glucocorticoid effect on both memory enhancement as well as the reduction in c-Fos expression. These findings thus support the view that noradrenergic activation of the BLA might even be a prerequisite for reducing aIC activity during the postlearning consolidation period. However, NE administration into the BLA of HC control rats did not reduce aIC activity, indicating a critical interaction between noradrenergic activation and learning-associated neuronal activity. Further, a more extensive training session, which induces comparable memory as after NE administration, also did not reduce the number of pCREB-positive neurons within the aIC. In one experiment, we found that a 3-min training experience even increased the number of pCREB-positive neurons within the aIC, such that NE administration into the BLA appeared to block this training effect (23). However, this finding was not unequivocal, as in other experiments, NE administration into the BLA reduced the expression of pCREB and c-Fos, as well as their colocalization, within the aIC in the absence of any training-induced increase. Thus, these findings indicate that this reduction in aIC neuronal activity is specifically related to an altered emotional arousal state, induced by the posttraining NE treatment, and is not needed to create a strong long-term memory of the training experience per se.

To investigate how noradrenergic activation of the BLA might induce such a reduction in aIC neuronal activity, we examined the number GAD67-positive puncta at the somatic circumference of pCREB-expressing neurons. GAD67 is an enzyme that is critically involved in the activity-dependent synthesis of GABA (49); therefore, the level of GAD67 expression might reflect presynaptic release probability of GABA and inhibitory transmission (33). NE administration into the BLA induced a large and significant increase in the number of perisomatic GABAergic inhibitory contacts per pCREB-expressing neuron. Thus, these findings strongly suggest that noradrenergic activation of the BLA reduces aIC activity via a dynamic up-regulation of GABAergic inhibitory tone. It is well established that GABA-mediated synaptic inhibition is crucial in neural circuit operations (50) and that learning-dependent neural plasticity can induce a dynamic modulation of the number of GAD67-positive puncta (33). The BLA is known to project not only onto excitatory neurons within the cortex but also has an even stronger innervation onto parvalbumin and somatostatin-expressing interneurons (27). Importantly, direct pharmacological augmentation of GABAergic tone within the aIC with muscimol administration 1 h after the training trial enhanced object recognition memory, indicating that this suppression of aIC activity during the consolidation phase actively contributes to emotional memory enhancement. This finding alone is notable, as many previous studies have shown that posttraining administration of a GABAergic agonist into many other brain regions (e.g., the BLA and hippocampus) severely disrupts memory processing (51, 52).

These findings thus strongly suggest that the information storage process underlying emotional enhancement of object recognition memory by noradrenergic activation of the BLA might not be the same as that induced by stimulation of local consolidation processes within the aIC. A recent model proposed that emotional arousal triggers dynamically and temporally regulated shifts in large-scale brain network balance, enabling an organism to comprehensively reallocate its neural resources according to cognitive demands (17, 53). Arousing stimulation, by activating NE, will first rapidly strengthen BLA–aIC connectivity and increase salience network activity at the cost of the executive control network (16, 19, 54). This brain state facilitates the detection and integration of external salient stimuli during memory encoding (16, 19, 53). However, in a delayed period after the initial arousal exposure, resource allocation to these two networks reverses: The salience network is suppressed, and the executive control network will become active (17). Our finding of reduced aIC activity 1 h after the training experience is thus in concordance with this dynamic regulation of salience network activity. According to the model, this delayed shutting off of salience network activity requires the poststress release of glucocorticoid hormones (17, 22). However, our findings suggest that an augmented GABAergic inhibition induced by noradrenergic activation of the BLA might also be able to shut off salience network activity. Notably, the aIC works as a “switch” that determines the extent to which information is relayed back to sensory cortices for use by the central executive network before being processed in the internally oriented default-mode network (55). Thus, a potential functional implication of this delayed suppression of aIC activity by noradrenergic activation of the BLA would be a protection of the consolidation process from interference by new encoding through a disruption of attentional reorienting toward external salient stimuli (17, 53). This action might simultaneously promote ongoing long-term memory storage processes within default-mode network regions such as the perirhinal cortex, which is also importantly involved in recognition memory (7).

In summary, the findings of the current study indicate that the effect of noradrenergic activation on enhancing object recognition memory is associated with a BLA-induced suppression of aIC activity during the postlearning consolidation period. These findings thus provide fundamental insight into the broader effects on brain network dynamics underlying emotional enhancement of memory.

## Materials and Methods

### Subjects.

Male Sprague-Dawley rats (280 to 320 g) from Charles River Breeding Laboratories were kept individually in a temperature-controlled (21 ± 1 °C) vivarium room (lights on: 7:00 AM to 7:00 PM) with ad libitum access to food and water. Training and testing were performed between 10:00 AM and 3:00 PM. All experimental procedures were in compliance with European Union Directive 2010/63/EU and were approved by the Institutional Animal Care and Use Committees of Radboud University, Nijmegen, and Sapienza University, Rome.

### Cannula Implantation.

Rats were anesthetized with ketamine (37.5 mg/kg, subcutaneous) and dexmedetomidine (0.25 mg/kg, subcutaneous). They further received the nonsteroidal analgesic carprofen (4 mg/kg), and 3 mL sterile saline. The rat was positioned in a stereotaxic frame (Kopf Instruments), and two stainless-steel guide cannulas (15 mm; 23 gauge; Component Supply Co/SKU Solutions) were implanted bilaterally with the cannula tips 2.0 mm above the BLA [AP, −2.8 mm from Bregma; mediolateral (ML), ±5.0 mm from midline; dorsoventral (DV), −6.5 mm from skull surface], aIC [AP, +1.0 mm; ML, ±5.5 mm; DV, −4.8 mm (below Bregma)], or pIC [AP, −2.0 mm; ML, ±5.8 mm; DV, −4.8 mm (below Bregma)] (56). Stylets (15-mm-long 00-insect dissection pins) were inserted into each cannula to maintain patency. After surgery, the rats were administered atipamezole hydrochloride (0.25 mg/kg, subcutaneous; Orion) to reverse anesthesia. The rats were allowed to recover for a minimum of 10 d before training.

### Viral Injection and Tracing.

AAV2/1-CB7-mCherry (105544-AAV1) and pAAV-CAG-GFP (37825-AAVrg) were obtained from Addgene. rAAV2-retro-CAG-tdTomato was generously provided by virus services in HHMI-Janelia Research Campus (Ashburn, VA). Viral titrations were 1 × 10^13^ gc/mL for AAV2/1-CB7-mCherry, 7 × 10^12^ gc/mL for pAAV-CAG-GFP, and 1.8 × 10^12^ gc/mL for rAAV2-retro-CAG-tdTomato. Viruses were microinjected unilaterally into the left hemisphere at a software-controlled rate of 10 nL/min with a 26-gauge 10-μL nanofil syringe. For anterograde tracing of BLA projections to the aIC, 10 rats were injected with 500 nL of AAV2/1-CB7-mCherry into the BLA (AP, −2.8 mm; ML, +5.1 mm; DV, −8.5 mm) (56). For retrograde tracing of BLA projections to the aIC, three rats were injected with 200 nL of rAAV2-retro-CAG-tdTomato into the agranular aIC [AP, +2.5 mm; ML, +4.4 mm; DV, −6.7 mm (below Bregma)]. For retrograde tracing of BLA projections to the pIC, two rats were injected with 300 nL of rAAV2-retro-CAG-tdTomato into the agranular pIC [AP, −2.0 mm; ML, +6.0 mm; DV, −7.6 mm (below Bregma)] and two rats with 500 nL of pAAV-CAG-GFP into the granular/dysgranular pIC [AP, −2.0 mm; ML, +6.2 mm; DV, −7.0 mm (below Bregma)]. After the injection, the needle was left in place for another 10 min.

After 2 to 3 wk, rats were anesthetized with sodium pentobarbital and perfused transcardially with ice-cold 0.1 M phosphate-buffered saline (PBS) (pH 7.4), followed by ice-cold 4% paraformaldehyde (PFA) in 0.1 M PBS (pH 7.4). Brains were postfixed for 1 h in 4% PFA and cryoprotected in 30% sucrose. Forty-micrometer-thick coronal sections were cut and stained with DAPI (1:5,000, D1306, Thermo Fisher Scientific). Images were acquired with an automated stitching fluorescent microscope (DMI 6000B, Leica Microsystems) using a 10× or 20× objective. Images were examined with FIJI software (NIH, version 1.0), and the anatomical location of fluorescent labeling within the regions of interest was verified with the Paxinos and Watson rat brain atlas (56).

### Object Recognition Task.

The experimental apparatus was a gray open-field box (in centimeters: 40 w × 40 d × 40 h) with the floor covered with sawdust, positioned in a dimly illuminated room (60 l×). The objects to be discriminated were white glass light bulbs (6-cm diameter, 11-cm length) and transparent glass vials (5.5-cm diameter, 5-cm height) secured to the floor of the box with Velcro tape. Each rat was placed individually in the experimental apparatus and allowed to explore two identical objects (A1 and A2) for either 3 or 10 min. To avoid the presence of olfactory trails, sawdust was stirred and the objects were thoroughly cleaned with 70% ethanol in between trials. Some animals were killed 1 h after training and drug treatment for tissue collection; other rats were tested for retention 24 h after the training trial. On the 24-h retention test, one copy of the familiar object (A3) and a new object (B) were placed in the same location as stimuli during the training trial. All combinations and locations of objects were used in a balanced manner to reduce potential biases due to preference for particular locations or objects. The rat was placed in the experimental apparatus for 3 min, and the time spent exploring each object was recorded. Rats’ behavior during training and the retention test was recorded with a video camera positioned above the experimental apparatus. Videos were analyzed off-line by a trained observer who was blind to treatment condition. Exploration of an object was defined as pointing the nose to the object at a distance of <1 cm and/or touching it with the nose (5). Turning around, climbing, or sitting on an object per se was not included in exploration times. A discrimination index was calculated as the difference in time exploring the novel and familiar object, expressed as the ratio of the total time spent exploring both objects (i.e., [Time Novel − Time Familiar/Time Novel + Time Familiar] × 100%).

### Local Drug Administration.

NE [(±)-1-(3,4-dihydroxyphenyl)-2-aminoethanol hydrochloride; Sigma-Aldrich] was dissolved in sterile saline and administered bilaterally into the BLA (1.0 μg in 0.2 μL), aIC (2.5 μg in 0.5 μL), or pIC (2.5 μg in 0.5 μL) immediately after the training trial (or to HC control rats) (5). The β-adrenoceptor antagonist propranolol hydrochloride (Sigma-Aldrich) was also dissolved in saline and administered bilaterally into the BLA (0.3 μg in 0.2 μL), aIC (0.75 μg in 0.5 μL), or pIC (0.75 μg in 0.5 μL) immediately after training (5). The GABAergic agonist muscimol (0.07 μg in 0.5 μL, 3-hydroxy-5-aminomethyl-isoxazole, Research Biochemicals International) was dissolved in saline and administered bilaterally into the aIC 1 h after the training trial (57). Drug infusions were given via 30-gauge injection needles connected to 10-μL Hamilton microsyringes by polyethylene (PE-20) tubing. The injection needles protruded 2.0 mm beyond the cannula tips, and drug or an equivalent volume of saline control, at a rate of 0.4 μL/min, was infused by an automated syringe pump (Stoelting Co.). The injection needles were retained within the cannulas for an additional 20 s. All drug solutions were freshly prepared before each experiment.

### Immunohistochemistry.

Rats were anesthetized with sodium pentobarbital and perfused transcardially with ice-cold 0.1 M PBS (pH 7.4), followed by ice-cold 4% PFA in 0.1 M PBS (pH 7.4). Brains were postfixed in 4% PFA for 1 h and cryoprotected in 30% sucrose. Forty-micrometer-thick coronal sections were cut on a cryostat, collected in Tris-buffered saline (TBS) with 0.1% sodium azide and phosphatase inhibitors (20 mM sodium fluoride and 2 mM sodium orthovanadate) and stored at 4 °C. Three sections of the aIC (AP, +2.5 to +1.7 mm) and pIC (AP, −1.7 to −2.5 mm) were selected from each rat. Sections were permeabilized with 0.5% Triton X-100 (Sigma-Aldrich) in 0.1 M TBS with phosphatase inhibitors for 30 min, blocked with 5% donkey serum (017-000-1221, Jackson ImmunoResearch), 5% goat serum (10000c, Thermo Fisher Scientific), 1% BSA (Thermo Fisher Scientific), 1% glycine (Sigma-Aldrich), and 0.3% Triton X-100 for 1 h and then incubated overnight with primary antibodies [pCREB (Rabbit anti-pCREB [Ser-133]; 1:100, Cell Signaling 9198 L), c-Fos (Goat anti–c-Fos; 1:200, Santa Cruz sc-52-G), GAD67 (Mouse anti-GAD67; 1:250, Millipore MAB5406), and NeuN (Chicken anti-NeuN; 1:500, Millipore ABN 91)] in blocking buffer at room temperature (RT). Sections were subsequently washed in 0.1 M TBS with phosphatase inhibitors and incubated with fluorophore-conjugated secondary antibodies [Donkey anti-Rabbit Alexa Fluor 488 (1:200 A21206), Goat anti-Chicken Alexa Fluor 568 (1:200 A11041), Donkey anti-Goat Alexa Fluor 568 (1:1,000 A11057), Goat anti-Mouse Alexa Fluor 568 (1:500 A11031), Donkey anti-Rabbit Alexa Fluor 647 (1:200 A31573), Thermo Fisher Scientific] for 3 h at RT. Sections were mounted, air-dried, and coverslipped with Fluorosave Mounting Medium.

### Imaging and Quantification.

Fluorescent images were acquired at 40× magnification using a fluorescent microscope (Zeiss Axio Imager 2, Carl Zeiss Microscopy), and image processing was performed in FIJI (NIH, version 1.0). For each aIC and pIC subdivision (agranular, dysgranular, and granular), a squared area (200 × 200 μm) was selected to cover layers II/III. The number of neurons showing expression of pCREB, c-Fos, GAD67, or NeuN was counted manually. For colocalization of c-Fos with pCREB and pCREB with GAD67 immunoreactivity, the number of colocalized nuclei was counted in each aIC subdivision, then averaged per subdivision for each rat. For quantitative analysis of GAD67-positve puncta, images were acquired at 63× magnification, and the number of GAD67-positive puncta per pCREB-positive nucleus was counted manually in layers II/III of each aIC subdivision and then averaged per subdivision for each image.

### Western Blot.

Three coronal slices of 250-μm thickness of the aIC (AP, +2.5 to +1.7 mm) and pIC (AP, −1.7 to −2.5 mm) of HC control rats were cut on a cryostat. Bilateral punches of the agranular aIC and pIC (thus six punches per region of interest) were acquired with a 1.0-mm brain puncher and snap frozen in isopentane on dry ice. Protein extracts were separated by sodium dodecyl sulfate polyacrylamide gel electrophoresis, transferred on polyvinylidene difluoride membranes (Bio-Rad, 170–4156), and probed with antibodies against the β_1_-adrenoceptor (Rabbit, 1:1,000, Invitrogen PA1-049) and β_2_-adrenoceptor (Mouse, 1:100, Santa Cruz, sc-271322) and glyceraldehyde 3-phosphate dehydrogenase (GAPDH, 1:1,000, Cell Signaling #2118) for normalization. Proteins were then detected with horseradish peroxidase–conjugated Goat anti-Mouse (1:50,000, Jackson ImmunoResearch Laboratories, 115–035-062) and Goat anti-Rabbit (1:50,000, Invitrogen, G21234). Proteins were revealed with Super Signal West Femto ECL (Thermo Fisher Scientific, 34095) and visualized with ChemiDoc Touch Imaging system (Bio-Rad).

### Statistics.

The discrimination index and object exploration time were analyzed with unpaired *t* tests. One-sample *t* tests were used to examine whether the discrimination index was different from zero (i.e., chance level). Immunohistochemistry data were analyzed with mixed-model ANOVAs with NE treatment and training as between-subject variable and IC subarea (aIC and pIC) or aIC subdivision (agranular, dysgranular, and granular aIC) as within-subject variable, followed by two-way ANOVAs for each subarea or subdivision separately. Post hoc analyses used unpaired and paired *t* tests. Western blot data were analyzed with paired *t* tests. *P* < 0.05 was accepted as statistical significance.

## Supplementary Material

Supplementary File

## Data Availability

All study data are included in the article and/or *SI Appendix*.
