# Supplementary material for: Basolateral amygdala activation enhances object recognition memory by inhibiting anterior insular cortex activity
Source: Proc Natl Acad Sci U S A. 2022 May 27;119(22):e2203680119. doi: 10.1073/pnas.2203680119 (PMC9295787; doi:10.1073/pnas.2203680119)
Supplement: Supplementary File [file pnas.2203680119.sapp.pdf]

PNAS
www.pnas.org

Supplementary Information for
Basolateral amygdala activation enhances object recognition memory by inhibiting anterior insular cortex activity

Yan-Fen Chen, Qi Song, Paola Colucci, Federica Maltese, Cristina Siller-Pérez, Karina Prins, James L. McGaugh, Erno J. Hermans, Patrizia Campolongo, Nael Nadif-Kasri and Benno Roozendaal

James L. McGaugh
Email: james.mcgaugh@uci.edu

Benno Roozendaal
Email: Benno.Roozendaal@radboudumc.nl

This PDF file includes:

Figures S1 to S7
Tables S1 to S5

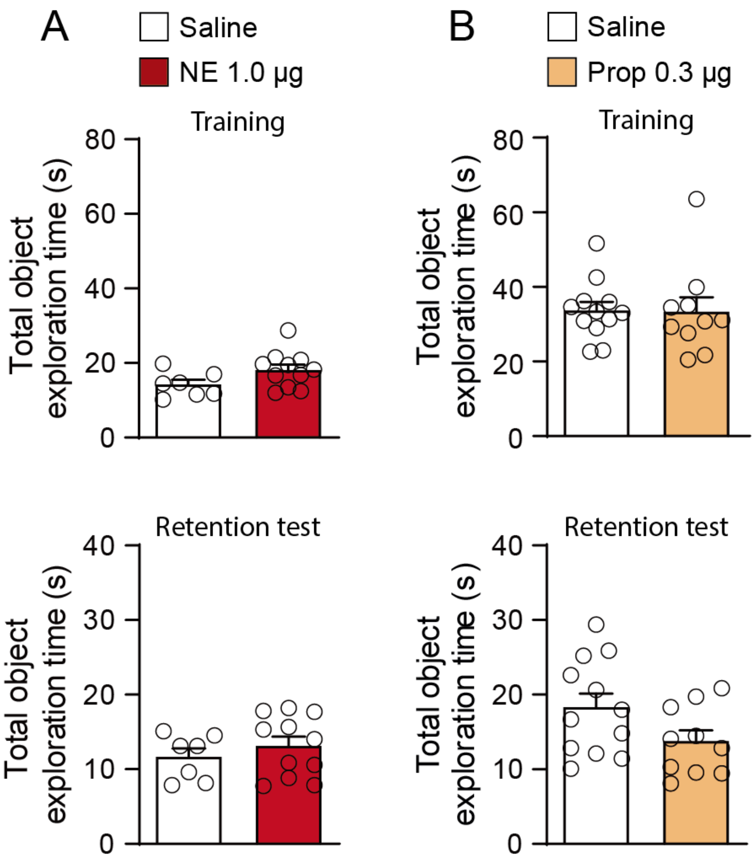

Fig. S1. Total object exploration time (in seconds) during the training trial and retention test of rats included in the experiments shown in Fig. 1 (see text for details). (*A*) Total object exploration time during the 3-min training trial (saline: *n* = 7 rats; NE: *n* = 11 rats; unpaired *t*-test: t_{16} = -1.88, *P* = 0.08) and retention test (t_{16} = -0.83, *P* = 0.42) of rats given immediate posttraining infusions of NE (1.0 µg in 0.2 µl) or saline into the BLA. (*B*) Total object exploration time during the 10-min training trial (saline: *n* = 12 rats; propranolol: *n* = 10 rats; t_{20} = 0.07, *P* = 0.95) and retention test (t_{20} = 1.80, *P* = 0.09) of rats given immediate posttraining infusions of propranolol (0.3 µg in 0.2 µl) or saline into the BLA. Data are expressed as mean ± SEM. Dots in the different graphs represent individual data points.

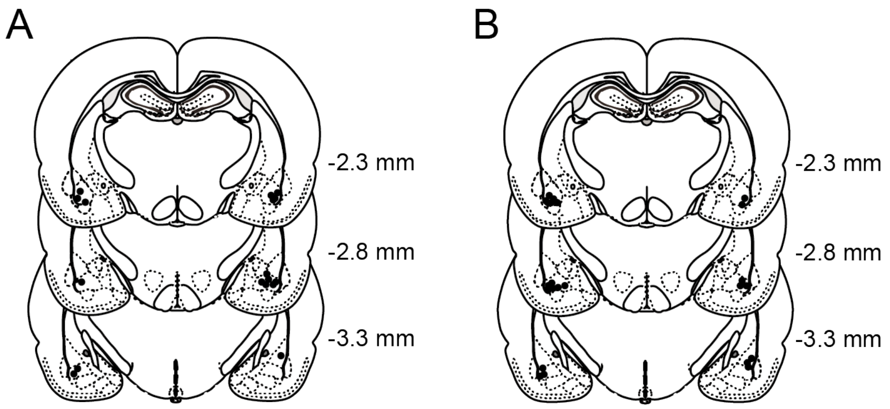

Fig. S2. Infusion sites in the BLA of rats included in Fig. 1. (*A*) Infusion sites in the BLA of all rats included in Fig. 1*C.* (*B*) Infusion sites in the BLA of all rats included in Fig. 1*D.*

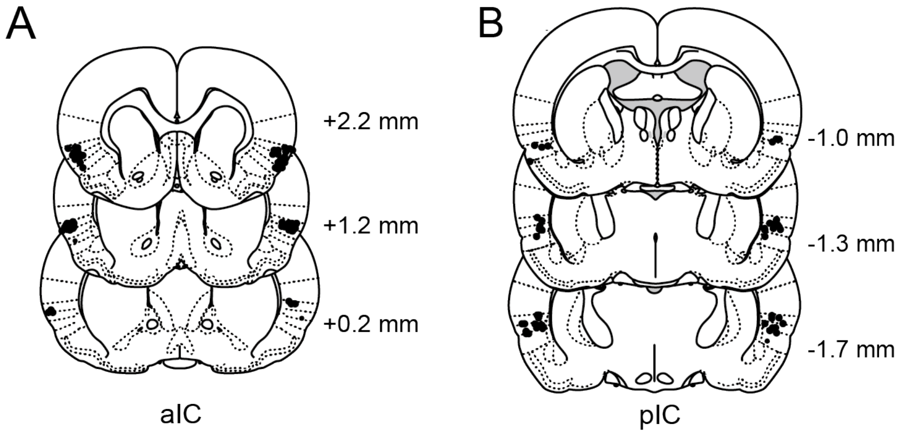

Fig. S3. Infusion sites in the aIC and pIC of rats included in Fig. 2. (*A*) Infusion sites in the aIC of all rats included in Fig. 2*C.* (*B*) Infusion sites in the pIC of all rats included in Fig. 2*D.*

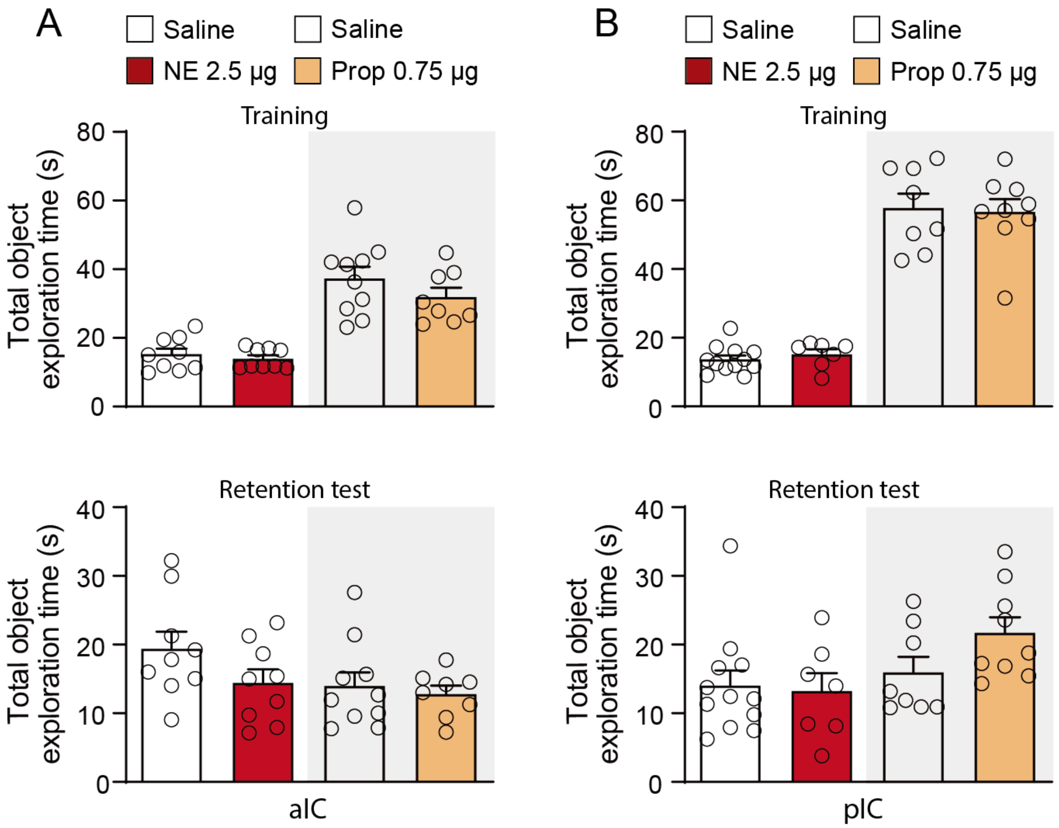

Fig. S4. Total object exploration time (in seconds) during the training trial and retention test of rats included in the experiments shown in Fig. 2 (see text for details). (*A*) Total object exploration time during the training trial (saline: *n* = 9 rats; NE: *n* = 9 rats; unpaired *t*-test: t_{16} = 0.70, *P* = 0.50; saline: *n* = 10 rats; propranolol: *n* = 8 rats; t_{16} = 1.21, *P* = 0.24) and retention test (NE: t_{16} = 1.57, *P* = 0.14; propranolol: t_{16} = 0.46, *P* = 0.65) of rats given immediate posttraining infusions of NE (2.5 µg in 0.5 µl) or propranolol (0.75 µg in 0.5 µl) into the aIC. For the NE experiments rats were trained for 3 min for the propranolol experiment for 10 min. The retention test was always 3 min. (*B*) Total object exploration time during the training trial (saline: *n* = 12 rats; NE: *n* = 7 rats; t_{17} = -0.84, *P* = 0.41; saline: *n* = 8 rats; propranolol: *n* = 9 rats; t_{15} = 0.19, *P* = 0.85) and retention test (NE: t_{17} = 0.23, *P* = 0.82; propranolol: t_{15} = -1.79, *P* = 0.09) of rat given immediate posttraining infusion of NE (2.5 µg in 0.5 µl) or propranolol (0.75 µg in 0.5 µl) into the pIC.. Data are expressed as mean ± SEM. Dots in the different graphs represent individual data points.

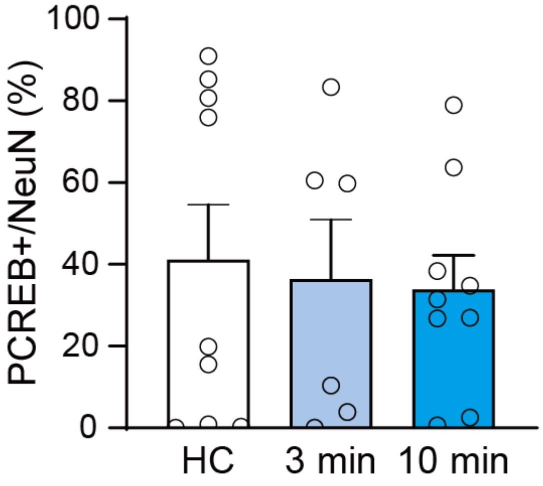

Fig. S5. Training on the object recognition task *per se* did not change the pCREB+/NeuN ratio within the aIC. Rats were trained on the object recognition task for either 3 or 10 min, or served as home cage (HC) control, and were sacrificed 1 h later for analysis of pCREB/NeuN immunofluorescence within all three subdivisions of the aIC (HC: *n* = 9 rats; 3 min: *n* = 6 rats, 10 min: *n* = 9 rats, one-way ANOVA: $F_{2,21}$ = 0.10, *P* = 0.90). Data are expressed as mean ± SEM. Dots in the graph represent individual data points.

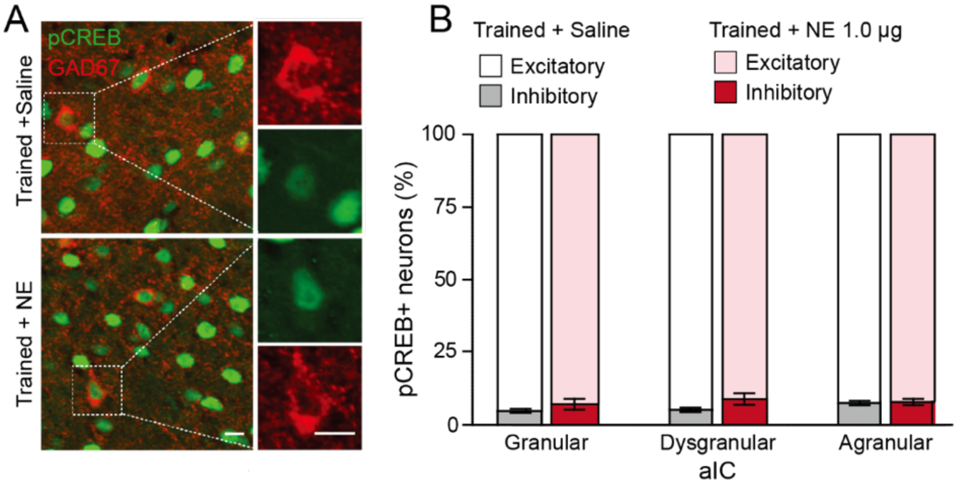

Fig. S6. Noradrenergic activation of the BLA after object recognition training did not change the percentage of pCREB-positive inhibitory versus excitatory neurons. The percentage of pCREB-positive inhibitory neurons was calculated by determining the number of neurons that show colocalized immunofluorescence for pCREB and GAD67, divided by the total number of pCREB-positive neurons. (*A*) Representative images showing GAD67 (red) and pCREB (green) immunofluorescence in the aIC. Scale bar: 10 µm. (*B*) Percentage of pCREB-positive inhibitory neurons in NE and saline-treated trained rats (saline: n = 6 rats; NE: n = 8 rats, mixed-model ANOVA: NE treatment: $F_{1,12}$ = 2.25, P = 0.16; subdivision: $F_{2,24}$ = 2.67, P = 0.09; NE treatment x subdivision: $F_{2,24}$ = 2.00, P = 0.16).

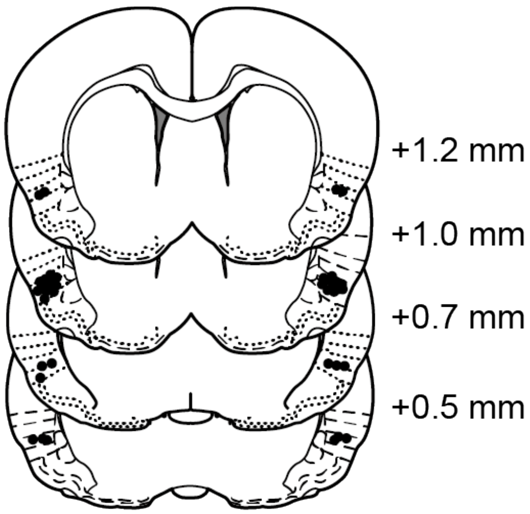

Fig. S7. Infusion sites in the aIC of all rats included in Fig. 5*G*.

Table S1. Total object exploration time during training of rats included in the experiment shown in Fig. 4*C*

	Saline into BLA<br>*n* = 5	NE (1.0 µg) into BLA<br>*n* = 5
Object exploration time training (s)	18.8 ± 4.6	18.2 ± 3.3

Data are shown as mean ± SEM

Table S2. Total object exploration time during training of rats included in the experiment shown in Fig. S5

	Home cage<br>*n* = 9	3 min training<br>*n* = 6	10 min training<br>*n* = 9
Object exploration time training (s)	-	12.8 ± 0.8	30.2 ± 2.9

Data are shown as mean ± SEM

Table S3. Total object exploration time during training of rats included in the experiment shown in Fig. 5*B-D*

	Saline into BLA<br>*n* = 9	NE (1.0 µg) into BLA<br>*n* = 6
Object exploration time training (s)	17.9 ± 1.5	20.6 ± 2.8

Data are shown as mean ± SEM

Table S4. Total object exploration time during training of rats included in the experiment shown in Fig. 5*F*

	Saline into BLA<br>*n* = 5	NE (1.0 μg) into BLA<br>*n* = 3
Object exploration time training (s)	23.2 ± 3.8	17.4 ± 5.6

Data are shown as mean ± SEM

Table S5. Total object exploration time during training and retention test of rats included in the experiment shown in Fig. 5*G*

	Saline into aIC<br>*n* = 11	Muscimol (0.07 µg) into aIC<br>*n* = 10
Object exploration time training (s)	28.4 ± 2.3	32.2 ± 3.3
Object exploration time retention (s)	24.2 ± 1.7	31.5 ± 5.1

Data are shown as mean ± SEM
